# Micromagnetic Investigation of the Effect of Main-Phase Distribution on the Coercivity of Nd_2_Fe_14_B/Dy_2_Fe_14_B Exchange-Coupled Magnets

**DOI:** 10.3390/nano16181139

**Published:** 2026-09-10

**Authors:** Ying Yu, Qian Zhao, Haoran Wang, Qingkang Hu, Suo Bai, Guoping Zhao, Zhubai Li

**Affiliations:** 1 School of Science, Inner Mongolia University of Science and Technology, Baotou 014010, China; 2 School of Physics and Electronic Engineering, Sichuan Normal University, Chengdu 610101, China; 3 Rare Earth Industry Research Institute, Inner Mongolia University of Science and Technology, Baotou 014010, China

**Keywords:** Nd_2_Fe_14_B/Dy_2_Fe_14_B, main phase distribution, coercivity, magnetocrystalline anisotropy field

## Abstract

The spatial distribution of different hard magnetic phases is a key factor affecting the coercivity of dual-main-phase rare-earth permanent magnets. However, the underlying relationship between main-phase distribution and magnetization reversal behavior in Nd_2_Fe_14_B/Dy_2_Fe_14_B magnets remains unclear. In this work, micromagnetic simulations based on MuMax3 and OOMMF are performed to systematically investigate the effects of main-phase spatial arrangement on the magnetic properties and magnetization reversal mechanisms of Nd_2_Fe_14_B/Dy_2_Fe_14_B exchange-coupled magnets. First, single-phase Nd_2_Fe_14_B and Dy_2_Fe_14_B models are constructed to clarify the intrinsic magnetic characteristics of the two phases. The calculated demagnetization curves show that, although the magnetocrystalline anisotropy field *H_A_* of Nd_2_Fe_14_B is lower than that of Dy_2_Fe_14_B, its higher saturation magnetization *M_S_* results in a slightly larger anisotropy constant *K*, according to *K* = $\frac{1}{2}$ *µ*_0_·*H_A_*·*M_S_*. Nevertheless, Dy_2_Fe_14_B exhibits a stronger resistance to magnetization reversal, as reflected by its higher nucleation field *H_N_* and coercivity *H_C_*. This indicates that the resistance to magnetization reversal is more directly associated with *H_A_* than with *K* alone. Subsequently, three types of exchange-coupled dual-main-phase Nd_2_Fe_14_B/Dy_2_Fe_14_B magnet models, including cubic, cylindrical, and sandwich structures, are constructed with identical size fractions of the two phases to investigate the influence of phase spatial distribution on magnetization reversal behavior. The calculated results demonstrate that placing the Dy_2_Fe_14_B phase in the outer region leads to higher *H_N_* and *H_C_* than the reverse phase arrangement, owing to its higher *H_A_*, which strengthens the resistance against magnetization reversal. Further analysis of the in-plane magnetic-moments and angular distributions reveals that magnetic-moment deviation is initially activated in the Nd_2_Fe_14_B region with lower *H_A_*, followed by gradual propagation through exchange coupling at the phase interface. This effect of phase spatial distribution is not limited to Nd_2_Fe_14_B/Dy_2_Fe_14_B exchange-coupled magnets. In Nd_2_Fe_14_B/La_2_Fe_14_B and Nd_2_Fe_14_B/SmCo exchange-coupled magnets, placing the phase with the higher *H_A_* in the outer region likewise results in higher *H_N_* and *H_C_* than the reverse phase arrangement. In addition, for all the dual-main-phase exchange-coupled magnets considered above, the coercive field decreases with increasing magnet size. These findings provide theoretical insights into the regulation of coercivity through spatial phase distribution in dual-main-phase rare-earth permanent magnets.

## 1. Introduction

In 1991, Kneller and Hawig proposed the concept of exchange-coupled composite magnetic materials [1]. As one of the exchange-coupled magnetic phases in such materials [2,3,4,5], Nd_2_Fe_14_B has attracted extensive attention in both theoretical and experimental research [6,7,8]. owing to its high saturation magnetization and large maximum energy product, which have enabled its widespread applications in various fields, including wind power generation, intelligent manufacturing, new energy vehicles, and energy-efficient household appliances [9,10]. However, compared with Dy_2_Fe_14_B, Nd_2_Fe_14_B exhibits lower coercivity and inferior thermal stability [11], which limits its application in high-performance environments. Therefore, in industrial production, heavy rare-earth element Dy is commonly introduced into Nd_2_Fe_14_B magnets through partial substitution of Nd, effectively improving the coercivity and thermal stability of the magnets [12,13]. However, Dy_2_Fe_14_B suffers from lower saturation magnetization and higher material cost, making it unsuitable as a complete replacement for Nd_2_Fe_14_B. Therefore, the structural design of exchange-coupled Nd_2_Fe_14_B/Dy_2_Fe_14_B dual-main-phase magnets that exploit the high saturation magnetization of Nd_2_Fe_14_B and the high coercivity of Dy_2_Fe_14_B to achieve a balance between coercivity and remanence while reducing Dy consumption remains an important research topic [14].

The spatial phase distribution is one of the key structural factors governing the magnetic properties of Nd_2_Fe_14_B/Dy_2_Fe_14_B exchange-coupled magnets [15]. Therefore, to clarify how the spatial distribution of the two phases regulates the critical fields and magnetization-reversal behavior of Nd_2_Fe_14_B/Dy_2_Fe_14_B exchange-coupled magnets, it is important to understand the role of their distinct intrinsic magnetic properties. It is well known that under an applied demagnetizing field, two critical fields associated with the reversal of magnetic moments, namely the nucleation field (defined as the negative value of the applied field at which the magnetic moments begin to deviate from their initial orientation) and the pinning field (defined as the negative value of the applied field before irreversible magnetization reversal occurs), are key parameters for evaluating the magnetic properties of permanent magnets. Although Dy_2_Fe_14_B possesses a higher magnetocrystalline anisotropy field *H_A_* than Nd_2_Fe_14_B, Nd_2_Fe_14_B exhibits a slightly larger anisotropy constant *K* because of its substantially higher saturation magnetization *M_S_*, as expressed by *K* = $\frac{1}{2}$ *µ*_0_·*H_A_*·*M_S_*. Therefore, under identical geometric dimensions and sizes, it is necessary to clarify whether the critical fields of single-phase Nd_2_Fe_14_B and Dy_2_Fe_14_B magnets are mainly governed by *H_A_* or *K*. Furthermore, the influence of magnet size on the magnetic properties of single-phase magnets should be systematically investigated. These studies on single-phase magnets provide the fundamental basis for understanding the underlying relationship between main-phase distribution and magnetization-reversal behavior in Nd_2_Fe_14_B/Dy_2_Fe_14_B dual-main-phase magnets.

In this study, the magnetic properties and magnetization-reversal processes of both single-phase magnets and exchange-coupled magnets are obtained by calculating demagnetization curves, in-plane magnetic moments, and angular distributions. First, single-phase Nd_2_Fe_14_B and Dy_2_Fe_14_B magnet models with identical dimensions, morphologies, and easy-axis orientations are constructed. The differences in magnetic properties and magnetization reversal behaviors between the two magnetic phases are investigated, providing a theoretical basis for the structural design of dual-main-phase magnets. Subsequently, for Nd_2_Fe_14_B/Dy_2_Fe_14_B exchange-coupled permanent magnets, cubic, cylindrical, and sandwich-structured models with different main-phase distributions are established while maintaining identical size fractions of the Nd_2_Fe_14_B and Dy_2_Fe_14_B phases. The effects of main-phase spatial distribution and size on the nucleation field and coercivity are systematically investigated. Furthermore, the evolution of magnetic domains and magnetization reversal processes under different phase distributions is analyzed by combining in-plane magnetic moment configurations and angular distribution. Since SmCo [16] and La_2_Fe_14_B [17] are also widely investigated permanent-magnet systems, Nd_2_Fe_14_B/SmCo and Nd_2_Fe_14_B/La_2_Fe_14_B exchange-coupled systems are further considered to examine the general applicability of the phase-distribution effect to different phase combinations. To verify the reliability of the simulation results and the general applicability of the conclusions, two micromagnetic simulation platforms, MuMax3 and OOMMF, based on micromagnetic theory are employed to investigate different structural models.

## 2. Calculation Models and Methods

Cubic, cylindrical, and sandwich-shaped permanent magnets are common geometries in practical applications. Therefore, different structural models in this study are simulated using MuMax3 (version 3.11) [18] and OOMMF (version 21a2) [19], according to the requirements of the respective geometries. Both packages are based on the Landau–Lifshitz–Gilbert (LLG) dynamic equation derived from micromagnetic theory [18,19,20,21,22,23]:

(1) $$\frac{dM}{dt}=-\left|\gamma \right|M\times H_{\mathrm{eff}}+\frac{\alpha }{M_{s}}\left(M\times \frac{dM}{dt}\right),$$

where ***M***, ***H****_eff_*, and *γ* represent the magnetization, effective field, and gyromagnetic ratio, respectively. The default value of *γ* is 2.211 × 10^5^ m·A^−1^·s^−1^. *M_S_* denotes the saturation magnetization, and α is the dimensionless damping coefficient. The experimental values of the damping coefficient α generally range from 0.01 to 0.1; however, it does not affect the equilibrium state of the system. Therefore, α is set to 0.5 in the simulations to reduce the computational time and energy dissipation. The effective field is defined as follows:

(2) $$H_{eff}=-{\mu_{0}}^{-1}\frac{\partial E}{\partial M}.$$

According to the Brown equation [20,24,25], the average energy density *E* is a function of the magnetization ***M***:

(3) $$E=A\left(r\right){\left[\frac{\nabla M}{M_{S}}\right]}^{2}-K\left(r\right)\frac{{\left(M\cdot n\right)}^{2}}{{M_{S}}^{2}}-\mu_{0}M\cdot H-\frac{1}{2}\mu_{0}H_{d}\left(r\right)\cdot M,$$

where ***H*** and ***H****_d_* (***r***) represent the external magnetic field and the demagnetizing field, respectively, and ***n*** is the unit vector along the easy axis direction ***e***. *A* and *K* denote the exchange energy constant and the volume anisotropy constant, respectively. The four terms at the right side of Equation (3) are in turn the exchange, anisotropy, applied field (Zeeman) and magnetostatic (demagnetization) energies.

**Figure 1:**
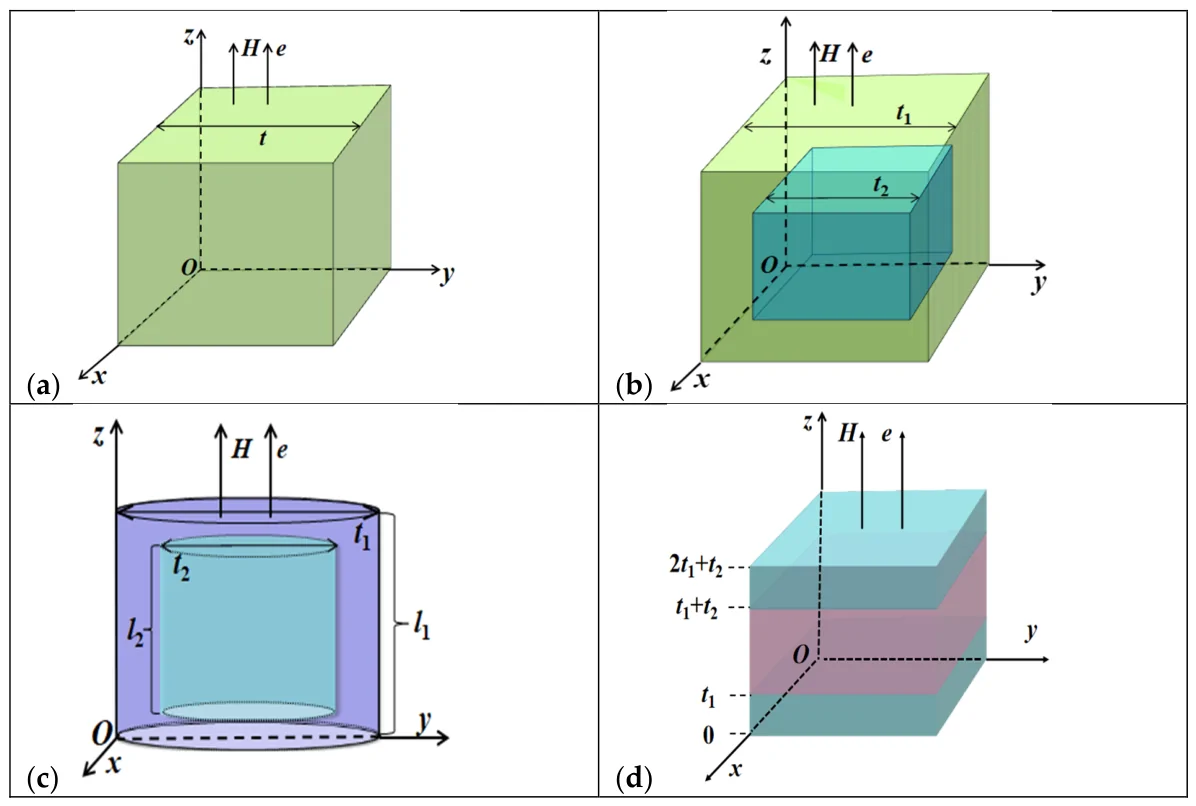
(color online): Computational models of Nd_2_Fe_14_B/Dy_2_Fe_14_B magnetic structures with the easy axis aligned along the *z*-axis. (**a**) represents the single-phase cubic Nd_2_Fe_14_B or Dy_2_Fe_14_B magnet; the remaining subfigures correspond to Nd_2_Fe_14_B/Dy_2_Fe_14_B exchange-coupled permanent magnets, specifically: (**b**) nested cubic structure; (**c**) nested cylindrical structure; and (**d**) cubic sandwich structure.

The two micromagnetic simulation software packages used in this work, MuMax3 [18] and OOMMF [19], are selected according to the requirements of different structural models. MuMax3 provides GPU-accelerated computation and high computational efficiency, making it suitable for relatively large three-dimensional models, whereas OOMMF offers flexible script-based modeling and good applicability to various two- and three-dimensional geometries. Therefore, MuMax3 is mainly used for the cylindrical and nested structural models, whereas OOMMF is employed for the sandwich-structured models. For all models constructed in this work, different colors are used in the model illustrations in Figure 1 to distinguish different magnetic phases. First, single-phase Nd_2_Fe_14_B and Dy_2_Fe_14_B cubic magnet models are constructed using the MuMax3 micromagnetic simulation software, as shown in Figure 1a. Subsequently, nested cubic and nested cylindrical structures of high-performance Nd_2_Fe_14_B/Dy_2_Fe_14_B exchange-coupled permanent magnets are designed using MuMax3. Finally, a cubic sandwich structure is established using the OOMMF micromagnetic simulation software to examine whether the observed phase-distribution effect is applicable to a different geometrical configuration. Based on the same nested geometric configurations, additional exchange-coupled Nd_2_Fe_14_B/La_2_Fe_14_B and Nd_2_Fe_14_B/SmCo models are constructed to further examine the generality of the phase-distribution effect. For the nested cubic and cylindrical dual-main-phase models, the two phases are arranged in inner–outer configurations with approximately equal phase volumes. The cubic sandwich models adopted a symmetric outer-layer/central-layer/outer-layer configuration with an exact 1:1 volume ratio between the total outer and central phase. For the additional Nd_2_Fe_14_B/La_2_Fe_14_B and Nd_2_Fe_14_B/SmCo models, the same nested cubic geometrical configurations and approximately equal phase-volume conditions as those used for the corresponding Nd_2_Fe_14_B/Dy_2_Fe_14_B models are adopted, with only the magnetic-phase composition changed. For each material combination, two opposite phase-distribution configurations are constructed by interchanging the inner and outer phases. For all models, the coordinate origin *O* is defined at the vertex of the bottom surface for cubic models or at an arbitrary point on the circumference of the bottom surface for the cylindrical models. A Cartesian coordinate system *O*–*xyz* is established with the *z*-axis perpendicular to the bottom surface, where the *z* = 0 plane corresponds to the *x*–*y* plane. The easy axis *e* and the applied magnetic field *H* are both aligned along the *z*-axis, perpendicular to the *x*–*y* reference plane. Thus, the easy axis and the applied magnetic field have the same orientation along the *z*-axis. For the nested cubic model, the geometric centers of the inner and outer cubes coincide, as shown in Figure 1b. For the nested cylindrical model, the central axes and geometric centers of the inner and outer cylinders coincide, as shown in Figure 1c. The OOMMF simulation model consists of a cubic sandwich structure, as shown in Figure 1d. For the single-phase cubic model, *t* denotes the side length. For the nested cubic model, *t*_1_ and *t*_2_ denote the outer and inner side lengths, respectively. For the nested cylindrical model, *t*_1_ and *t*_2_ denote the outer and inner diameters, respectively, while *l*_1_ and *l*_2_ denote their corresponding axial lengths. The geometric parameters of the nested structures are selected to obtain approximately equal volumes of the two magnetic phases. For the sandwich structure, the two outer layers have identical thicknesses, and their total thickness 2*t*_1_ equals the thickness of the central layer *t*_2_, satisfying 2*t*_1_ = *t*_2_.

In this work, Nd_2_Fe_14_B and Dy_2_Fe_14_B are considered as the two main phases of the Nd_2_Fe_14_B/Dy_2_Fe_14_B exchange-coupled permanent magnets. Additional Nd_2_Fe_14_B/La_2_Fe_14_B and Nd_2_Fe_14_B/SmCo exchange-coupled systems are considered to further examine the generality of the phase-distribution effect. The intrinsic magnetic parameters of the magnetic phases, including the exchange stiffness constant *A* and magnetocrystalline anisotropy constant *K*, are determined based on the following considerations. Assuming that the relevant phases possess the same crystal structure and point-group symmetry, the ratio of their exchange stiffness constants *A* is equal to the ratio of their corresponding Curie temperatures *T_C_* [26]. Therefore, the exchange stiffness constants of Dy_2_Fe_14_B and La_2_Fe_14_B are calculated from the reported exchange stiffness constant of Nd_2_Fe_14_B based on their respective Curie temperatures [26]. The magnetocrystalline anisotropy constant *K* of each phase is calculated from the reported magnetocrystalline anisotropy field *H_A_* and saturation magnetization *M_S_* [26] according to

(4) $$K=\frac{1}{2}\mu_{0}H_{A}M_{S},$$

where *H_A_*, *K*, and *M_S_* denote the magnetocrystalline anisotropy field, magnetocrystalline anisotropy constant, and saturation magnetization, respectively. The intrinsic parameters of Nd_2_Fe_14_B, Dy_2_Fe_14_B, La_2_Fe_14_B, and SmCo are summarized in Table 1. As shown in Table 1, compared with Nd_2_Fe_14_B, Dy_2_Fe_14_B exhibits a higher magnetocrystalline anisotropy field *H_A_* but a lower saturation magnetization *M_S_*, which results in a slightly lower magnetocrystalline anisotropy constant *K* according to Equation (4). For La_2_Fe_14_B, both *H_A_* and *K* are lower than those of Nd_2_Fe_14_B, whereas SmCo exhibits higher values of both *H_A_* and *K*.

The sample designations and corresponding geometric parameters of all single-phase and dual-main-phase models investigated in this work are summarized in Table 2. For the single-phase cubic magnets, the sample designation consists of the initial letters of the corresponding phase followed by the side length of the cube; for example, Nd-70 denotes a single-phase Nd_2_Fe_14_B cubic magnet with a side length of 70 nm. For the dual-main-phase nested cubic and nested cylindrical structures, the sample designation consists of the initial letters of the outer and inner main phases, followed by the corresponding outer characteristic dimension, namely, the outer side length for the nested cubic structures and the outer diameter for the nested cylindrical structures. For example, ND-70 denotes a dual-main-phase nested structure with Nd_2_Fe_14_B as the outer phase, Dy_2_Fe_14_B as the inner phase, and an outer characteristic dimension of 70 nm. For the cubic sandwich structures, the sample designation consists of the initial letters of the main phases arranged sequentially from the outer layer to the central layer and then to the outer layer, followed by the thickness of the outer layer. For example, NDN-6 denotes a sandwich structure with Nd_2_Fe_14_B/Dy_2_Fe_14_B/Nd_2_Fe_14_B arranged from the outer layer to the inner layer and an outer-layer thickness of 6 nm. The Nd_2_Fe_14_B/La_2_Fe_14_B and Nd_2_Fe_14_B/SmCo nested cubic models adopt the same geometric configurations and labeling convention as the Nd_2_Fe_14_B/Dy_2_Fe_14_B nested cubic models. The sample designations and geometric parameters defined above are used consistently throughout the manuscript. To achieve a balance between computational accuracy and simulation efficiency, different mesh sizes are employed for models constructed using the two simulation platforms. For the MuMax3-based models, a mesh size of 0.5 nm × 0.5 nm × 0.5 nm is adopted when the side length *t* of the single-phase cubic magnet or the outer side length (outer cylinder diameter) *t*_1_ is smaller than 100 nm. When *t*_1_ exceeded 100 nm, a mesh size of 2 nm × 2 nm × 2 nm is used. For the cubic sandwich structures constructed using OOMMF, a mesh size of 2 nm × 2 nm × 1 nm is employed. The selected mesh sizes are smaller than the Bloch wall widths [30,31,32] and are also smaller than the magnetostatic exchange lengths of the magnetic phases. The interfacial exchange coupling constant is set to the arithmetic mean of the exchange stiffness constants of the two adjacent magnetic phases. Accordingly, the interfacial exchange coupling constants for the Nd_2_Fe_14_B/Dy_2_Fe_14_B, Nd_2_Fe_14_B/La_2_Fe_14_B, and Nd_2_Fe_14_B/SmCo interfaces are 7.80 × 10^−12^ J·m^−1^, 7.35 × 10^−12^ J·m^−1^, and 9.85 × 10^−12^ J·m^−1^, respectively.

## 3. Results and Discussion

### 3.1. Demagnetization Processes of Single-Phase Nd_2_Fe_14_B and Dy_2_Fe_14_B Magnets

#### 3.1.1. Demagnetization Curve

**Figure 2:**
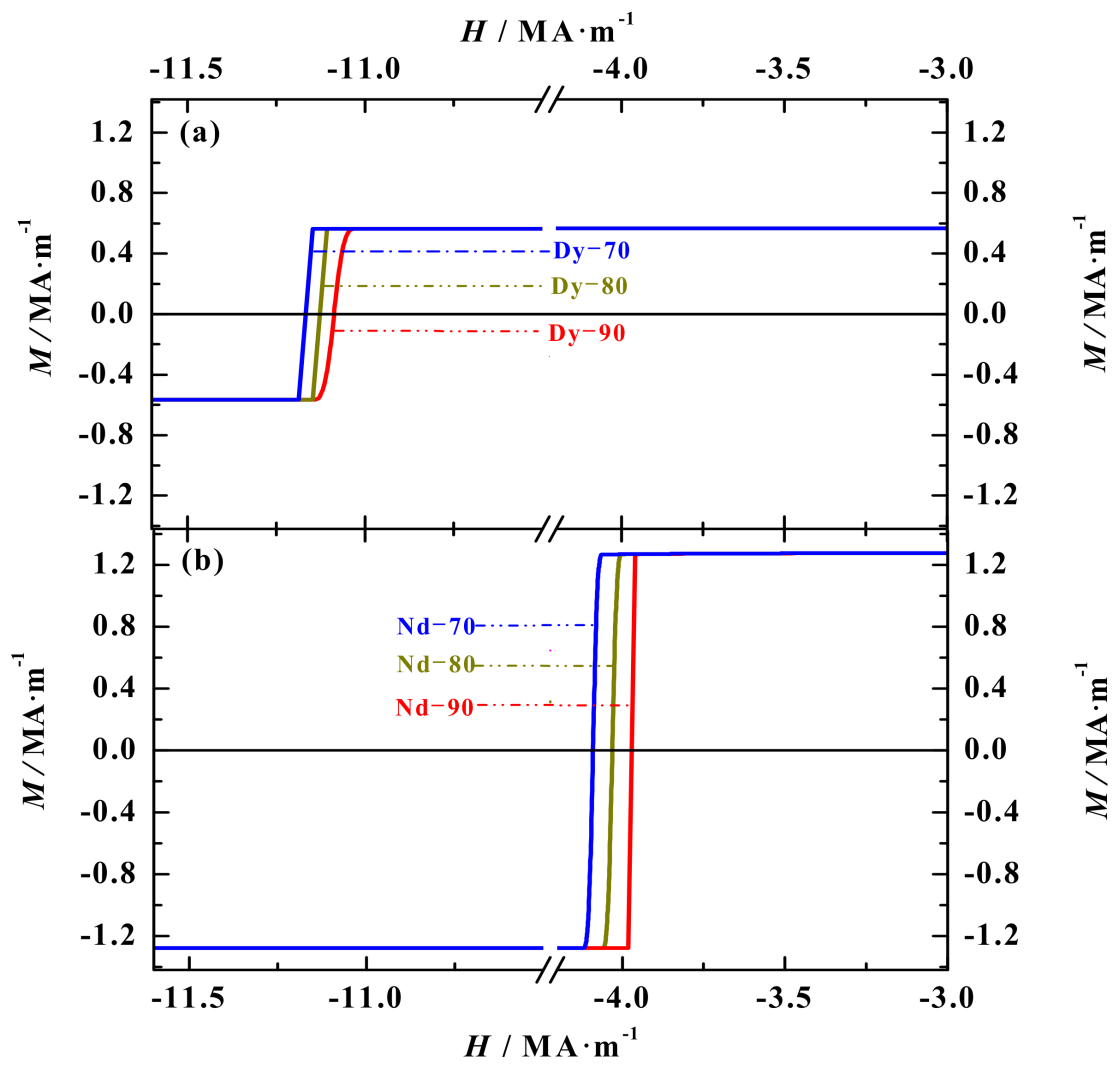
(color online): Demagnetization curves of single-phase (**a**) Dy_2_Fe_14_B and (**b**) Nd_2_Fe_14_B cubic magnets with the easy axes aligned along the *z*-axis. The labels “70,” “80,” and “90” represent the side lengths of the cubic magnets in nm.

Before investigating the effect of phase distribution on Nd_2_Fe_14_B/Dy_2_Fe_14_B exchange-coupled magnets, the intrinsic magnetic properties of the individual phases should be clarified. The key magnetic parameters characterizing the magnetic properties of permanent magnets, such as the nucleation field *H_N_*, pinning field *H_P_*, and coercivity *H_C_*, can be obtained from the demagnetization curves. Figure 2 presents the demagnetization curves of single-phase Dy_2_Fe_14_B (Figure 2a) and Nd_2_Fe_14_B (Figure 2b) magnets with different sizes. The corresponding model types, geometric parameters, and sample IDs are defined in Table 2. The nucleation field *H_N_*, pinning field *H_P_*, and coercivity *H_C_* obtained from the demagnetization curves are summarized in Table 3. For all single-phase magnets, *H_C_* is equal to *H_P_*, indicating that the coercivity mechanism is governed by domain-wall pinning. The size dependence of the critical fields can also be observed from the results for different magnet sizes. As the side length increases from 70 to 90 nm, the *H_N_* and *H_C_* of Nd_2_Fe_14_B decrease from 2.21 and 4.08 MA·m^−1^ to 1.61 and 3.96 MA·m^−1^, respectively, while those of Dy_2_Fe_14_B decrease from 10.79 and 11.15 MA·m^−1^ to 10.59 and 11.07 MA·m^−1^, respectively. This behavior can be attributed to the enhanced internal demagnetizing field in larger magnets, which facilitates the formation of local reversed regions and promotes the deviation of magnetic moments from their initial orientations. For identical dimensions, Dy_2_Fe_14_B consistently exhibits higher *H_N_* and *H_C_* than Nd_2_Fe_14_B (Table 3), demonstrating its stronger resistance against magnetization reversal. This difference is closely related to the magnetocrystalline anisotropy fields *H_A_* of the two magnetic phases. A larger *H_A_* increases the energy barrier required for magnetic moments to deviate from their initial easy-axis orientation, thereby enhancing *H_N_* and contributing to the enhancement of *H_C_*. According to Equation (4), *K* = $\frac{1}{2}$ *µ*_0_·*H_A_*·*M_S_*, although Dy_2_Fe_14_B exhibits a higher *H_A_* than Nd_2_Fe_14_B, its significantly lower saturation magnetization *M_S_* compensates for the increase in *H_A_*, resulting in a slightly lower magnetocrystalline anisotropy constant *K*.

To further examine this size-dependent behavior using the dimensions adopted in the subsequent nested cubic Nd_2_Fe_14_B/Dy_2_Fe_14_B exchange-coupled models, single-phase Nd_2_Fe_14_B and Dy_2_Fe_14_B magnets with side lengths of 55.5, 100, and 142 nm are also simulated. For Nd_2_Fe_14_B, the *H_N_* values are 2.47, 2.25, and 1.04 MA·m^−1^, and the corresponding *H_C_* values are 4.18, 3.92, and 3.80 MA·m^−1^, respectively; for Dy_2_Fe_14_B, the *H_N_* values are 10.95, 10.67, and 10.31 MA·m^−1^, and the corresponding *H_C_* values are 11.27, 11.03, and 10.87 MA·m^−1^, respectively. These results confirm that Dy_2_Fe_14_B possesses stronger resistance against magnetization reversal than Nd_2_Fe_14_B under identical geometrical conditions, and that the critical fields of both phases decrease with increasing magnet size.

**Figure 3:**
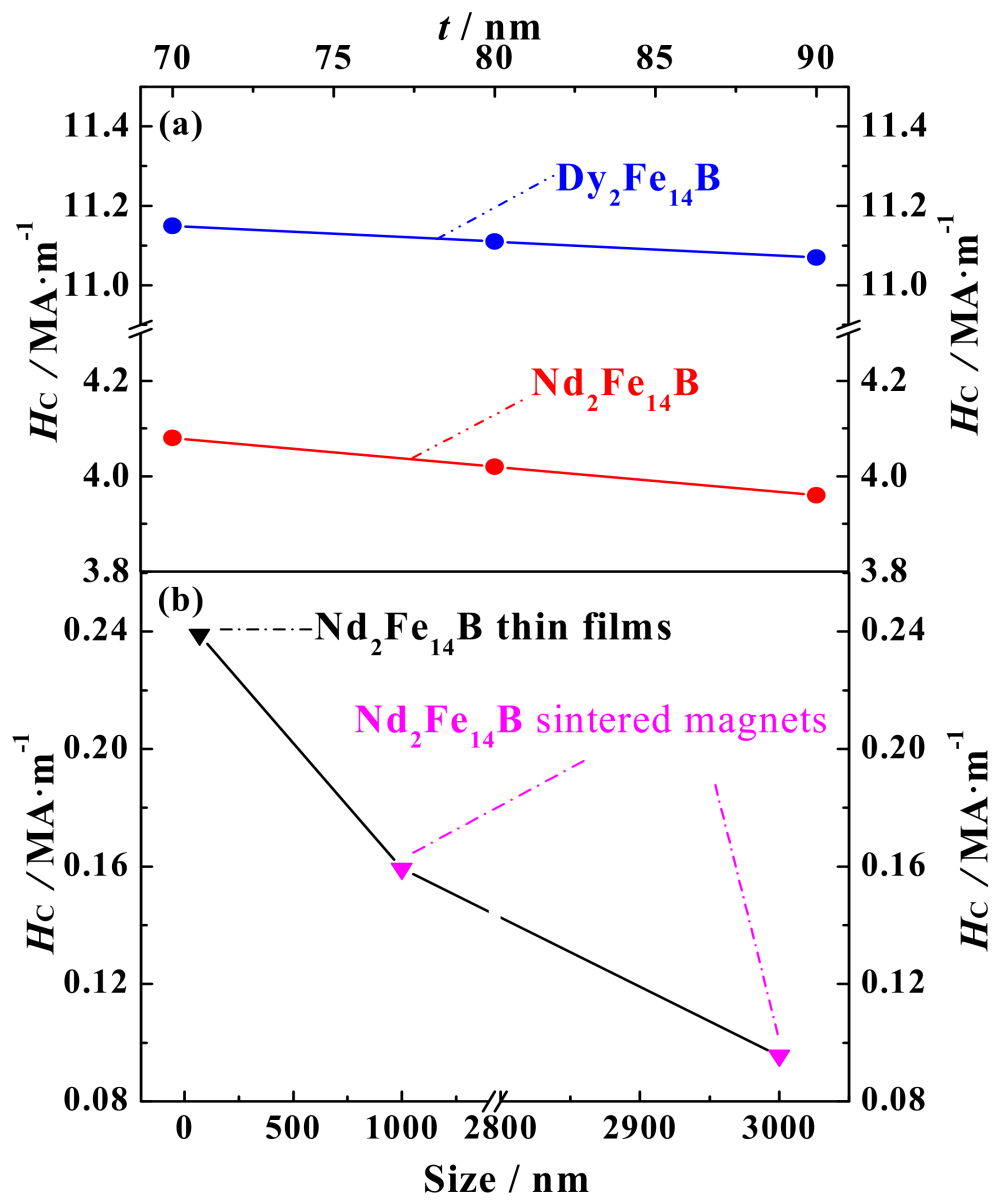
(color online): Comparison between theoretical and experimental coercivity data: (**a**) single-phase Nd_2_Fe_14_B and Dy_2_Fe_14_B cubic magnets with the easy axes aligned along the *z*-axis; (**b**) Nd_2_Fe_14_B thin films with the *c*-axis perpendicular to the film plane and sintered magnets [33]. In Figure (**a**), the symbol t in the theoretical calculation data represents the cube edge length, whereas in Figure (**b**), “Size” in the experimental data denotes the grain size.

The coercivities of both single-phase Nd_2_Fe_14_B and Dy_2_Fe_14_B magnets decrease with increasing magnet size. This size-dependent trend is qualitatively consistent with experimental observations reported for rare-earth permanent magnets, such as Nd_2_Fe_14_B [33,34] and SmCo_5_ [35] systems. Figure 3a presents the micromagnetic simulation results for Nd_2_Fe_14_B magnets, which are obtained from the data summarized in Table 3, whereas Figure 3b summarizes representative experimental data reported for Nd_2_Fe_14_B magnets. Specifically, the coercivity of Nd_2_Fe_14_B thin films with a grain size of approximately 70 nm is 0.23885 MA·m^−1^, while those of Nd_2_Fe_14_B sintered magnets with grain sizes of approximately 1000 and 3000 nm are 0.15924 and 0.09554 MA·m^−1^, respectively [33]. Experimentally, Nd_2_Fe_14_B magnets with larger grain sizes exhibit lower coercivity, indicating a similar size-dependent reduction in coercivity to that observed in the micromagnetic simulations. These experimental data are used only to demonstrate the qualitative trend and are not intended for direct quantitative comparison with the simulation results. The experimental samples and idealized simulation models differ significantly in microstructure, grain size, and reversal mechanisms; therefore, their absolute coercivity values are not directly comparable. Nevertheless, the experimentally observed decrease in coercivity with increasing characteristic size agrees well with the trend obtained from the simulations. Similar grain-size-dependent coercivity behavior has also been reported for SmCo_5_ permanent magnets [35].

#### 3.1.2. In-Plane Magnetic Moments

**Figure 4:**
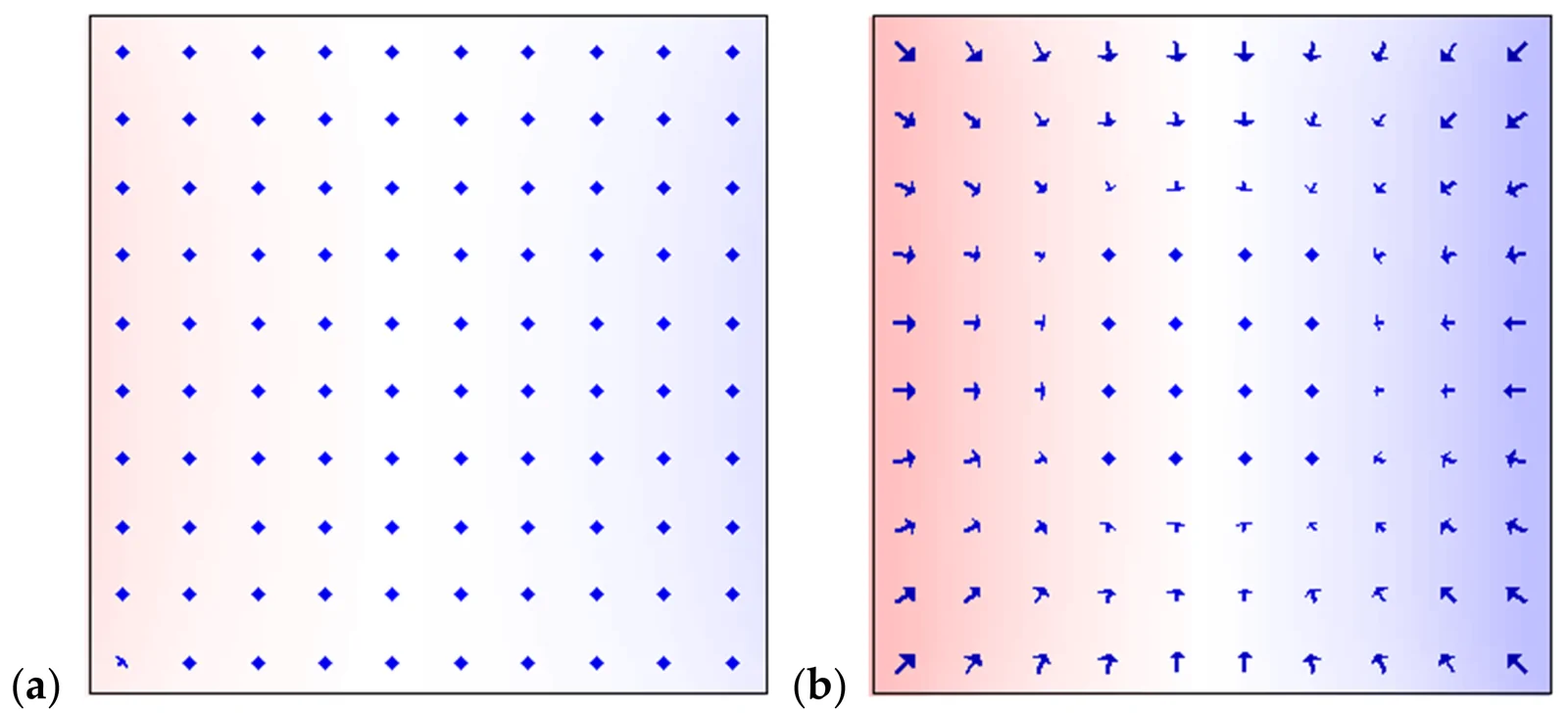

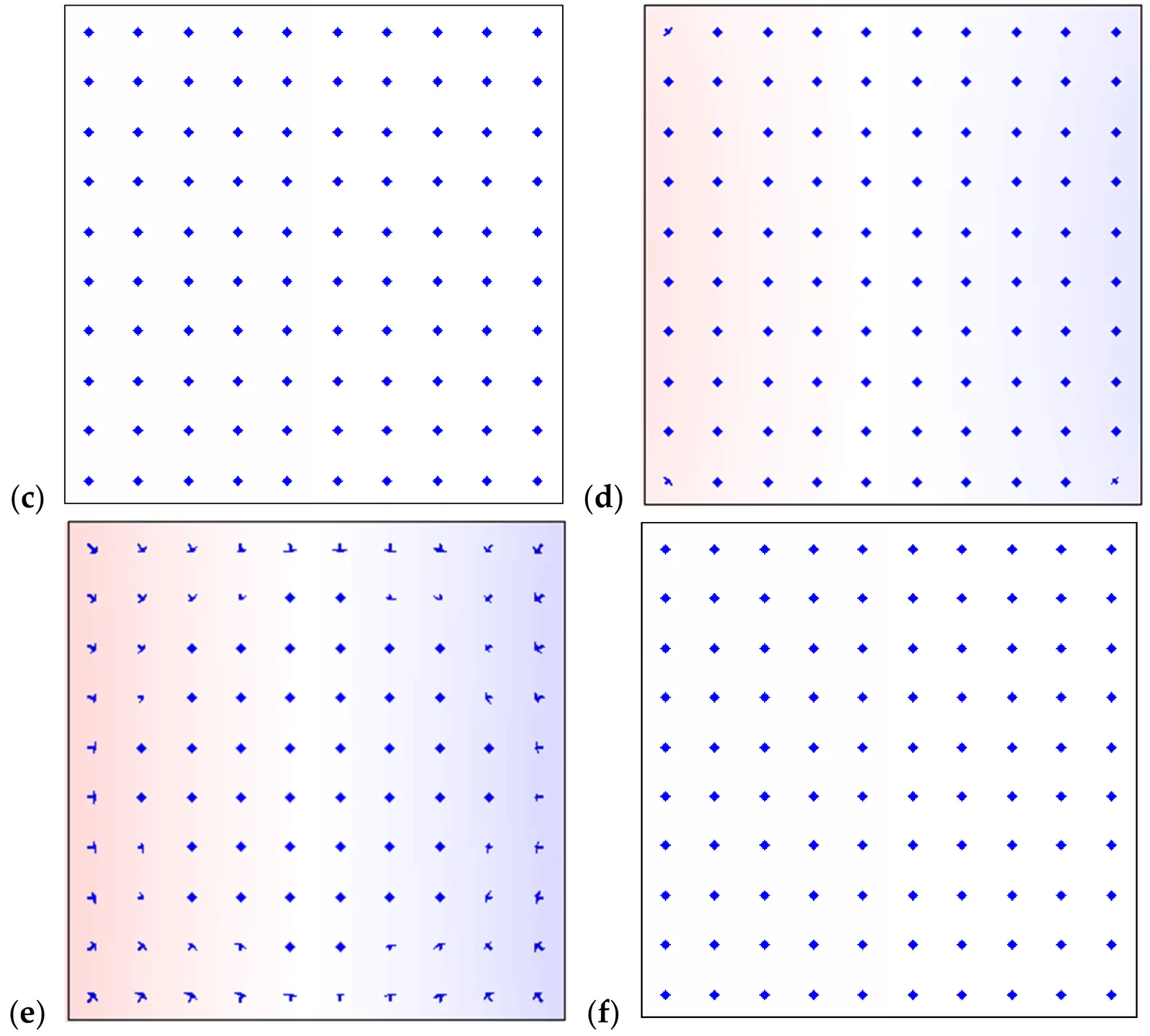
(color online): In-plane magnetization distributions during the evolution from nucleation to pinning in Nd_2_Fe_14_B (80 nm) and Dy_2_Fe_14_B (80 nm) magnets. For the Nd_2_Fe_14_B (80 nm) magnet: (**a**) surface magnetization distribution (*z* = 0 nm) at nucleation onset at *H*= −*H_N_* = −2.17 MA·m^−1^; (**b**) surface magnetization distribution (*z* = 0 nm) at pinning at *H* = −*H_P_* = −4.02 MA·m^−1^; (**c**) symmetric central position of the magnet (*z* = 40 nm) at pinning at *H* = −*H_P_* = −4.02 MA·m^−1^. For the Dy_2_Fe_14_B (80 nm) magnet: (**d**) surface magnetization distribution (*z* = 0 nm) at nucleation onset at *H* = −*H_N_* = −10.71 MA·m^−1^; (**e**) surface magnetization distribution (*z* = 0 nm) at pinning at *H* = −*H_P_* = −11.11 MA·m^−1^; (**f**) symmetric central position of the magnet (*z* = 40 nm) at pinning at *H* = −*H_P_* = −11.11 MA·m^−1^. For visualization, the magnetic-moment vectors are downsampled by a factor of 16, i.e., one vector is displayed for every 16 mesh points.

The evolution of in-plane magnetic moments under different external magnetic fields can provide further insight into the magnetic moment deviation and magnetization reversal mechanisms of permanent magnets, thereby explaining the behavior of critical fields (including the nucleation field and pinning field) from a microscopic perspective. Here, “in-plane” refers to the plane parallel to the *x*–*y* plane, i.e., the plane perpendicular to the *z*-axis, as illustrated in the model schematic in Figure 1. Figure 4 illustrates the evolution of in-plane magnetic moments during the process from nucleation to pinning for single-phase cubic Nd_2_Fe_14_B (Nd-80) and Dy_2_Fe_14_B (Dy-80) magnets with their easy axes aligned along the *z*-axis and a side length of 80 nm. All in-plane magnetic moment configurations are presented sequentially from bottom to top. Before nucleation, all magnetic moments remain aligned with the easy axis, i.e., the positive *z*-axis direction, without deviation, corresponding to the positively saturated magnetization state. When the demagnetizing field reaches the nucleation point *H* = −*H_N_*, the initial deviation of magnetic moments occurs near the surface corner region of the Nd_2_Fe_14_B magnet at *z* = 0 nm, indicating the onset of magnetic moments deviation, whereas the magnetic moments around the surface center remain essentially unchanged (Figure 4a). This behavior originates from the strong accumulation of surface magnetic charges at the edges and corners of the magnet, which generates a larger local demagnetizing field and causes the local effective field to first reach the threshold for magnetic moment deviation, resulting in the preferential deviation of magnetic moments near the corners. Under a stronger reverse magnetic field, when the demagnetizing field reaches the pinning point where *H* = −*H_P_*, the deviation of surface magnetic moments in the Nd_2_Fe_14_B magnet reaches its maximum, and the magnetic domain size expands to its maximum value (Figure 4b). However, even at the pinning stage, the magnetic moments at the center of the Nd_2_Fe_14_B surface (*z* = 0 nm, Figure 4b) and those at the symmetric central position of the magnet (*z* = 40 nm, Figure 4c) remain aligned without deviation. With a further increase in the demagnetizing field, irreversible magnetization reversal occurs, and all magnetic moments in the Nd_2_Fe_14_B magnet completely rotate to the negative *z*-axis, corresponding to the negatively saturated state. Similarly, for the Dy_2_Fe_14_B (Dy-80) magnet, the evolution of in-plane magnetic moments is analyzed during the magnetization reversal process. Compared with Nd-80, the magnetic moments at the surface corners of Dy_2_Fe_14_B (*z* = 0 nm) begin to deviate only under a much higher demagnetizing field where *H* = −*H_N_*, indicating the initiation of magnetic domain formation (Figure 4d). When the demagnetizing field increases to the pinning point where *H* = −*H_P_*, the surface magnetic moments of Dy_2_Fe_14_B reach their maximum deviation (Figure 4e). Meanwhile, the magnetic moments at the surface center and the symmetric central position (*z* = 40 nm, (Figure 4f)) remain unchanged.

For the Nd_2_Fe_14_B and Dy_2_Fe_14_B single-phase magnets with other identical dimensions (e.g., 70 and 90 nm), the evolution of the in-plane magnetic moments is qualitatively consistent with that observed for the 80 nm models. Under identical size and other geometric conditions, the Nd_2_Fe_14_B single-phase magnet exhibits lower nucleation, pinning, and coercive fields than the Dy_2_Fe_14_B magnet. More interestingly, at the corresponding critical fields, the magnetic moments in Nd_2_Fe_14_B exhibit larger angular deviations from the external magnetic-field direction, which is also the easy-axis direction in this study. This indicates that, compared with Nd_2_Fe_14_B, the higher magnetocrystalline anisotropy field *H_A_* of Dy_2_Fe_14_B provides stronger resistance to magnetic-moment deviation during magnetization reversal. During the demagnetization process, particularly between the nucleation and pinning fields, the evolution of the angular deviation of magnetic moments with the applied field in the two single-phase magnets is further examined through the angular distribution analysis presented below.

#### 3.1.3. Angular Distribution

**Figure 5:**
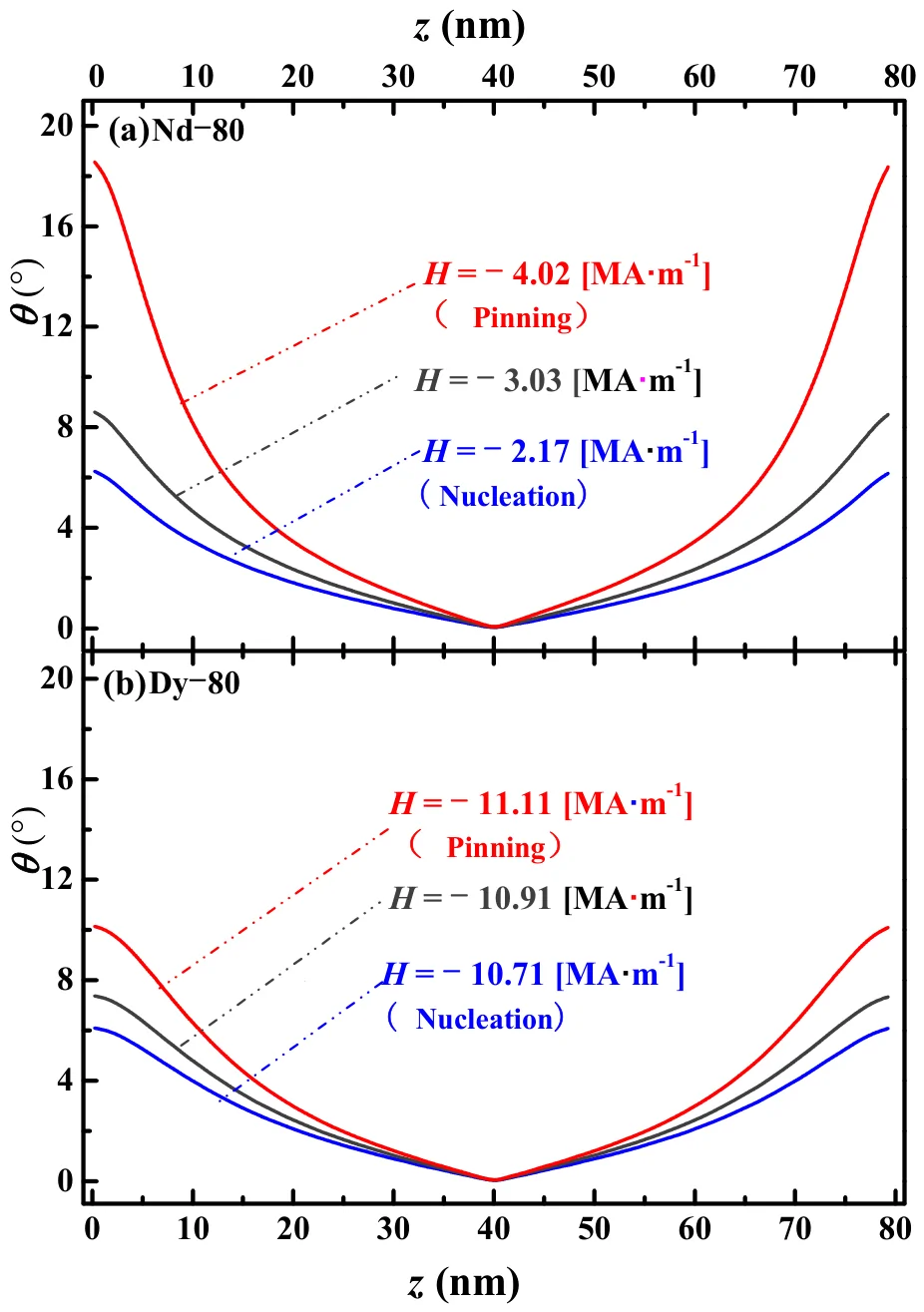
(color online): Angular distributions of the two single-phase magnets with a cube side length of 80 nm: (**a**) Nd-80 and (**b**) Dy-80. The angular values represent the average angle within the corresponding *z*-direction slice.

The angular distribution can clearly describe the deviation behavior of magnetic moments under an applied magnetic field and simultaneously reflect the evolution of magnetic domains. The angular distribution *θ*(*z*) is defined as the spatial distribution of the angle *θ* between the magnetic moments and the applied magnetic field *H* along the thickness direction (*z*-axis) of the magnet under a given external magnetic field. Figure 5 presents the angular distributions of cubic single-phase Nd_2_Fe_14_B (Nd-80) and Dy_2_Fe_14_B (Dy-80) magnets with their easy axes aligned along the *z*-axis and a side length of 80 nm during the process from nucleation to pinning. The angular distributions presented in Figure 5 cover a *z*-range of 0–80 nm with an interval of 0.5 nm. At each selected *z* position, the corresponding angular distribution value represents the average angle of magnetic moments within the corresponding slice. Each slice has a thickness of 0.5 nm along the *z*-axis, a cross-sectional area of 80 nm × 80 nm parallel to the *x*–*y* plane, and therefore represents a three-dimensional size within the magnet. For single-phase Nd_2_Fe_14_B and Dy_2_Fe_14_B magnets with different sizes, the angular distributions exhibit similar variation trends. Therefore, single-phase Nd_2_Fe_14_B (Nd-80) and Dy_2_Fe_14_B (Dy-80) magnets with a cubic side length of 80 nm are selected for detailed analysis, as shown in Figure 5. Owing to the symmetry of the models (see Figure 1a), the angular distributions of all magnets are symmetric with respect to the center of the cubic magnet.

The results in Figure 5 demonstrate that the number of magnetic domains in both single-phase Nd_2_Fe_14_B and Dy_2_Fe_14_B magnets remains two during the process from nucleation to pinning. Here, the number of magnetic domains is determined from the *θ*(*z*) angular distribution in Figure 5 by identifying the inflection points in the *θ*(*z*) curve, where the region between two adjacent inflection points corresponds to one magnetic domain. The magnetic domains are identified from the angular difference Δ*θ* between adjacent inflection points in the *θ*(*z*) curve, while the corresponding spatial difference Δ*z* represents the magnetic-domain size along the *z*-axis [36,37,38]. As shown in Figure 5a, for the Nd-80 magnet, before nucleation, the angle *θ* between the magnetic moments and the applied magnetic field is 0°, corresponding to the positively saturated magnetization state. At the nucleation point, *H* = −*H_N_* = −2.17 MA·m^−1^. At this stage, the angular difference is 6.18°, corresponding to a spatial domain size Δ*z* of 39 nm. With increasing demagnetizing field, the deviation angle of magnetic moments gradually increases, and the angular difference correspondingly increases. When the demagnetizing field reaches *H* = −3.03 MA·m^−1^, the angular difference increases to 8.54°. When the demagnetizing field reaches the pinning point *H* = −*H_P_* = −4.02 MA·m^−1^, the angular difference further increases to 18.45°. With a further increase in the demagnetizing field, irreversible magnetization reversal occurs, and all magnetic moments in the Nd_2_Fe_14_B magnet rotate completely to *θ* = 180° with respect to the applied field, reaching the negatively saturated state. For the Dy-80 magnet, as shown in Figure 5b, the initial angular difference at the nucleation point *H* = −*H_N_* = −10.71 MA·m^−1^ is 6.03°, with a corresponding spatial domain size of Δ*z* = 39 nm. With a further increase in the demagnetizing field, the angular difference Δ*θ* continues to increase. At *H* = −10.91 MA·m^−1^, the angular difference increases to 7.3°. When the demagnetizing field reaches the pinning point *H* = −*H_P_* = −11.11 MA·m^−1^, the angular difference increases to 10.05°. The spatial domain size remains constant at Δ*z* = 39 nm throughout the process from nucleation to pinning.

### 3.2. Demagnetization Processes of Nested Cubic Nd_2_Fe_14_B/Dy_2_Fe_14_B Magnets

#### 3.2.1. Demagnetization Curve

**Figure 6:**
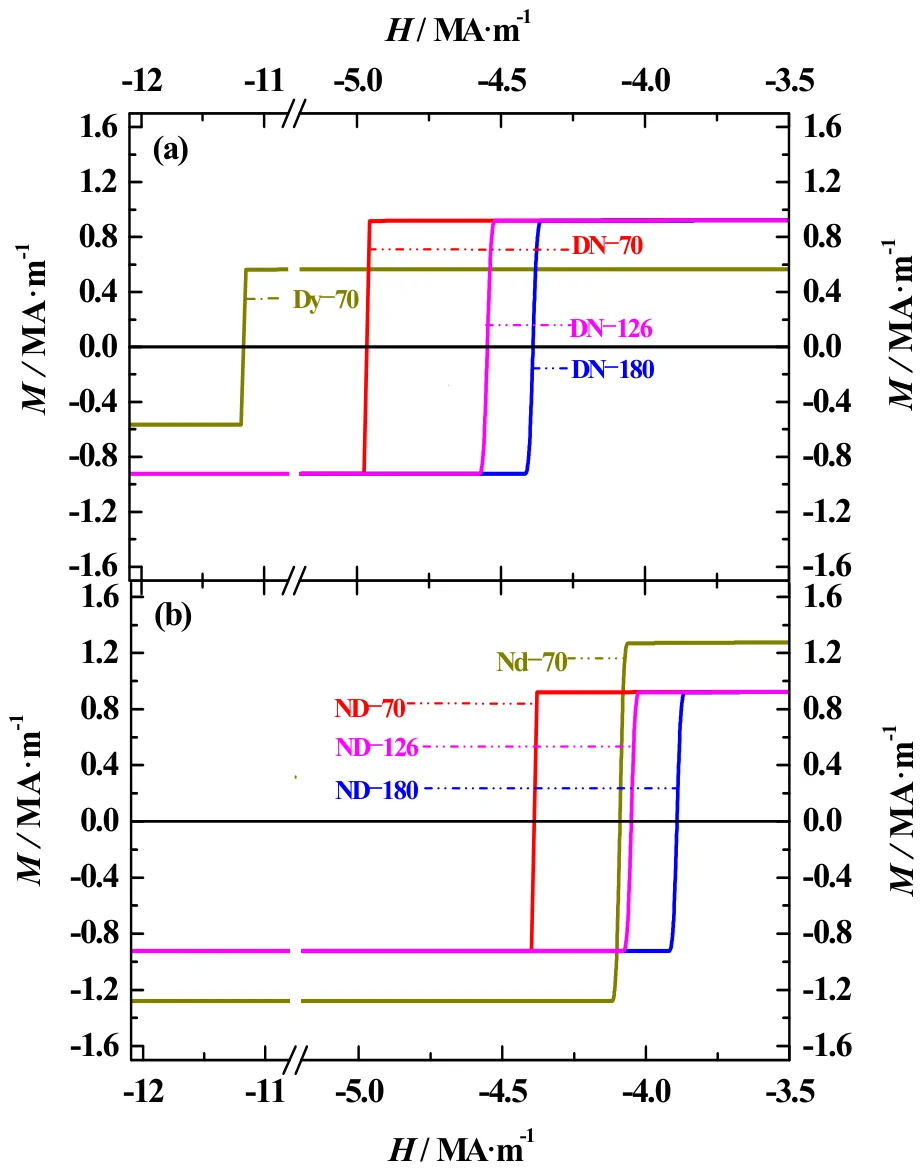
(color online): Demagnetization curves of Nd_2_Fe_14_B/Dy_2_Fe_14_B exchange-coupled nested cubic magnet structures with the easy axis aligned along the *z*-axis. Two configurations are compared: (**a**) Dy_2_Fe_14_B surrounding Nd_2_Fe_14_B (DN) and (**b**) Nd_2_Fe_14_B surrounding Dy_2_Fe_14_B (ND). The numbers denote the outer side lengths in nm.

Based on the analysis of the intrinsic parameters of Nd_2_Fe_14_B and Dy_2_Fe_14_B in Section 3.1, Nd_2_Fe_14_B possesses a lower magnetocrystalline anisotropy field *H_A_* than Dy_2_Fe_14_B, resulting in relatively low critical fields. To clarify how the spatial arrangement of the two main phases affects the critical fields and magnetization reversal behavior of Nd_2_Fe_14_B/Dy_2_Fe_14_B exchange-coupled magnets, Figure 6 presents the demagnetization curves of nested cubic models of these magnets with their easy axes aligned along the *z*-axis, as simulated using MuMax3. The corresponding model is illustrated in Figure 1b. Figure 6 shows that the outer and inner side lengths of these nested cubic magnets are 70 nm (55.5 nm), 126 nm (100 nm), and 180 nm (142 nm), respectively. In Figure 6a, the Dy_2_Fe_14_B phase is located outside the Nd_2_Fe_14_B phase (DN-70, DN-126, and DN-180), whereas in Figure 6b, the Nd_2_Fe_14_B phase is located outside the Dy_2_Fe_14_B phase (ND-70, ND-126, and ND-180). To compare the effect of Dy substitution for Nd in Nd_2_Fe_14_B on the magnetic properties of the magnets, single-phase cubic magnets with a side length of 70 nm, namely Dy_2_Fe_14_B (Dy-70) and Nd_2_Fe_14_B (Nd-70), are introduced into Figure 6a,b, respectively, as reference samples.

The nucleation fields *H_N_*, pinning fields *H_P_* and coercivities *H_C_* of Nd_2_Fe_14_B/Dy_2_Fe_14_B exchange-coupled magnets and single-phase Dy_2_Fe_14_B magnets extracted from the demagnetization curves in Figure 6 are summarized in Table 4. As shown in Table 4, *H_P_* is equal to *H_C_*, indicating that the coercivity is governed by a pinning mechanism. The DN-70 magnet exhibits an *H_N_* of 4.16 MA·m^−1^ and *H_C_* of 4.96 MA·m^−1^, whereas the ND-70 magnet has an *H_N_* of 3.07 MA·m^−1^ and *H_C_* of 4.38 MA·m^−1^. Thus, under the same size and phase fraction, placing Dy_2_Fe_14_B in the outer region leads to higher *H_N_* and *H_C_* than placing Nd_2_Fe_14_B in the outer region. Since the differences in remanence and maximum energy product among these structures are relatively small, only the remanence *M_r_* and maximum energy product (*BH*)_max_ of the DN-70 magnet are provided. The *M_r_* and (*BH*)_max_ values of DN-70 are 0.92 MA·m^−1^ and 267.07 kJ·m^−3^, respectively. For the same main-phase distribution, both *H_N_* and *H_C_* decrease with increasing magnet size. For the DN series, *H_N_* decreases from 4.16 to 3.36 MA·m^−1^, while *H_C_* decreases from 4.96 to 4.38 MA·m^−1^ as the outer side length of the magnet increases from 70 to 180 nm. A similar size-dependent decrease is observed for the ND series, where *H_N_* decreases from 3.07 to 2.61 MA·m^−1^ and *H_C_* decreases from 4.38 to 3.88 MA·m^−1^.

In addition, the demagnetization curves of single-phase Nd_2_Fe_14_B and Dy_2_Fe_14_B magnets with outer side lengths of 70, 126, and 180 nm are also calculated. At the same magnet size, both *H_N_* and *H_C_* exhibit the same increasing order: single-phase Nd_2_Fe_14_B, Nd_2_Fe_14_B/Dy_2_Fe_14_B magnets with the Nd_2_Fe_14_B phase located outside the Dy_2_Fe_14_B phase, Nd_2_Fe_14_B/Dy_2_Fe_14_B magnets with the Dy_2_Fe_14_B phase located outside the Nd_2_Fe_14_B phase, and single-phase Dy_2_Fe_14_B. Taking an outer side length of 70 nm as an example (see Table 3), *H_N_* and *H_C_* increase from 2.21 and 4.08 MA·m^−1^ for single-phase Nd_2_Fe_14_B to 10.79 and 11.15 MA·m^−1^, respectively, for single-phase Dy_2_Fe_14_B. These results indicate that, under the same size conditions, replacing Nd_2_Fe_14_B with Dy_2_Fe_14_B substantially increases both *H_N_* and *H_C_*.

#### 3.2.2. In-Plane Magnetic Moments

**Figure 7:**
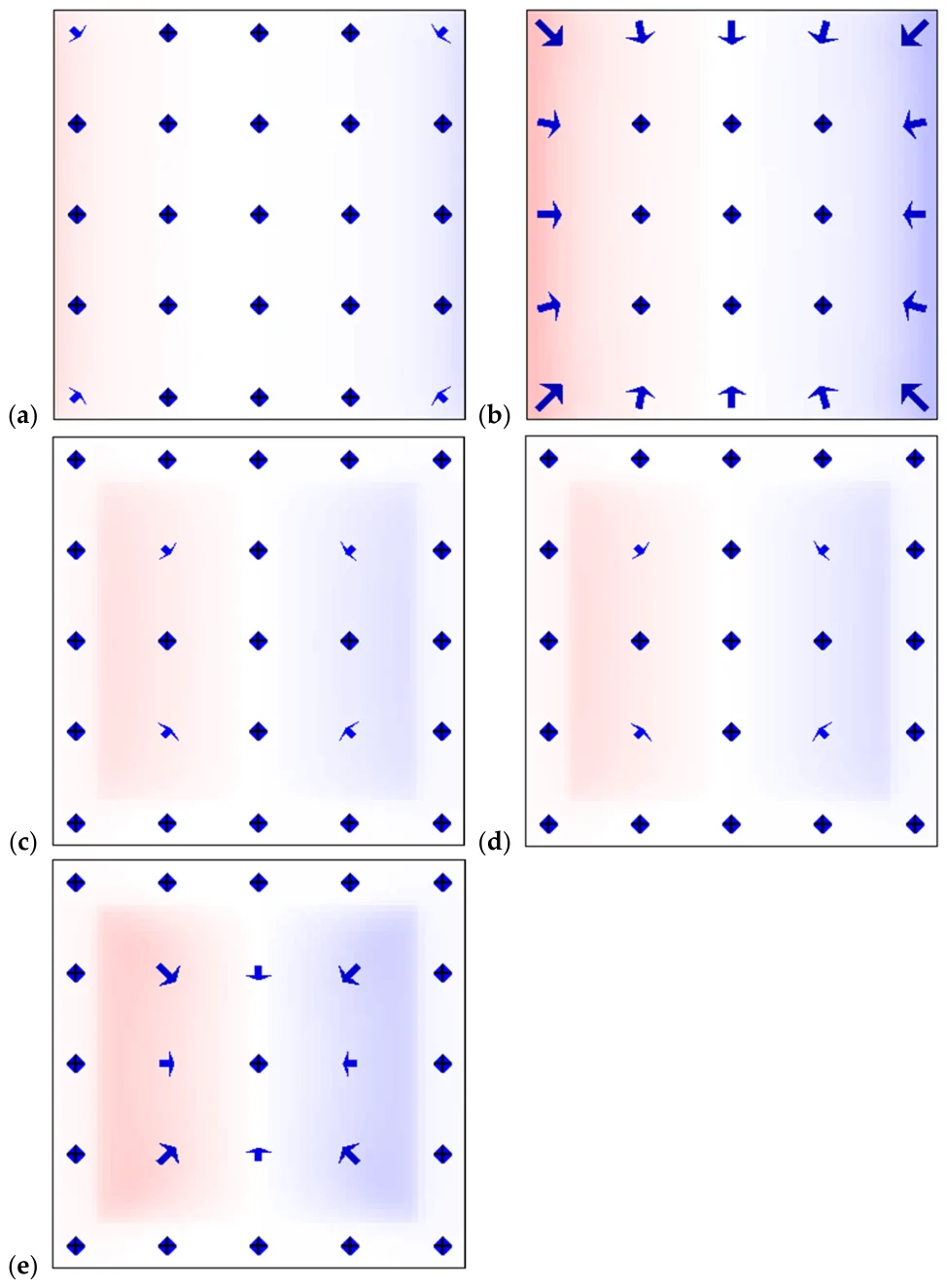
(color online): In-plane magnetic moment distributions of the ND-126 and DN-126 magnets at nucleation and pinning states. For the ND-126 magnet, (**a**) nucleation at *H* = −*H_N_* = −2.65 MA·m^−1^ on the Nd_2_Fe_14_B surface (*z* = 0 nm); (**b**) pinning at *H* = −*H_P_* = −4.04 MA·m^−1^ on the Nd_2_Fe_14_B surface (*z* = 0 nm). For the DN-126 magnet, (**c**) nucleation at *H* = −*H_N_* = −4.06 MA·m^−1^ in the Nd_2_Fe_14_B region at *z* = 17.99 nm; (**d**) interface at *H* = −4.40 MA·m^−1^ (*z* = 13 nm); (**e**) pinning at *H* = −*H_P_* = −4.54 MA·m^−1^ at *z* = 17.99 nm. For visualization, the magnetic-moment vectors are downsampled by a factor of 14, i.e., one vector is displayed for every 14 mesh points.

To further elucidate the mechanisms of magnetic-moment deviation and magnetization reversal from a microscopic perspective, Figure 7 presents the evolution of in-plane magnetic moments during the process from nucleation to pinning for two Nd_2_Fe_14_B/Dy_2_Fe_14_B exchange-coupled magnets with different spatial distributions of the main phases. Since the magnetic moment deviation behaviors of these exchange-coupled magnets with identical phase distributions but different sizes are essentially the same, only the in-plane magnetic moment evolution under different external magnetic fields of the two dual-main-phase structures with outer and inner side lengths of 126 nm and 100 nm, respectively, is discussed. As shown in Figure 1b, the thickness of the outer cubic shell phase is 13 nm. For both the ND-126 and DN-126 magnets, the magnetic-moment deviation characteristics before nucleation and after pinning are similar to those of the single-phase magnets described above. For the ND-126 magnet, in which the Nd_2_Fe_14_B phase is located in the outer region, the magnetic moments at the corners of the Nd_2_Fe_14_B surface (*z* = 0 nm) begin to deviate from their initial orientation at the nucleation point, *H* = −*H_N_* (Figure 7a). With decreasing applied magnetic field, the magnetic moments near the edges become increasingly tilted at the pinning point, *H* = −*H_P_* (Figure 7b). However, even at the pinning stage, the magnetic moments at the interface (*z* = 13 nm) and within the Dy_2_Fe_14_B phase remain unrotated. Similarly, the evolution of in-plane magnetic moments in the DN-126 magnet, in which the Dy_2_Fe_14_B phase is located in the outer region, is analyzed. At the nucleation point *H* = −*H_N_*, the magnetic moments also begin to deviate first within the Nd_2_Fe_14_B region. Since the magnetic moments within the region of *z* = 16–17.99 nm undergo rotation at this stage, the *z* = 17.99 nm plane is selected as a representative mesh plane to visualize this initial deviation, where the magnetic moments began to deviate around the center of the square region (Figure 7c). With decreasing applied field, when *H*= − 4.40 MA·m^−1^, the magnetic moments at the interface (*z* = 13 nm) also began to deviate around the center of the square region (Figure 7d). At the pinning point *H* = −*H_P_*, the deviation angle and the affected region of the magnetic moments around the center of the square region at *z* = 17.99 nm became enlarged, as shown in Figure 7e. However, even at this stage, the magnetic moments at the Dy_2_Fe_14_B surface (*z* = 0 nm) and inside the Dy_2_Fe_14_B phase remained completely aligned without deviation.

#### 3.2.3. Angular Distribution

**Figure 8:**
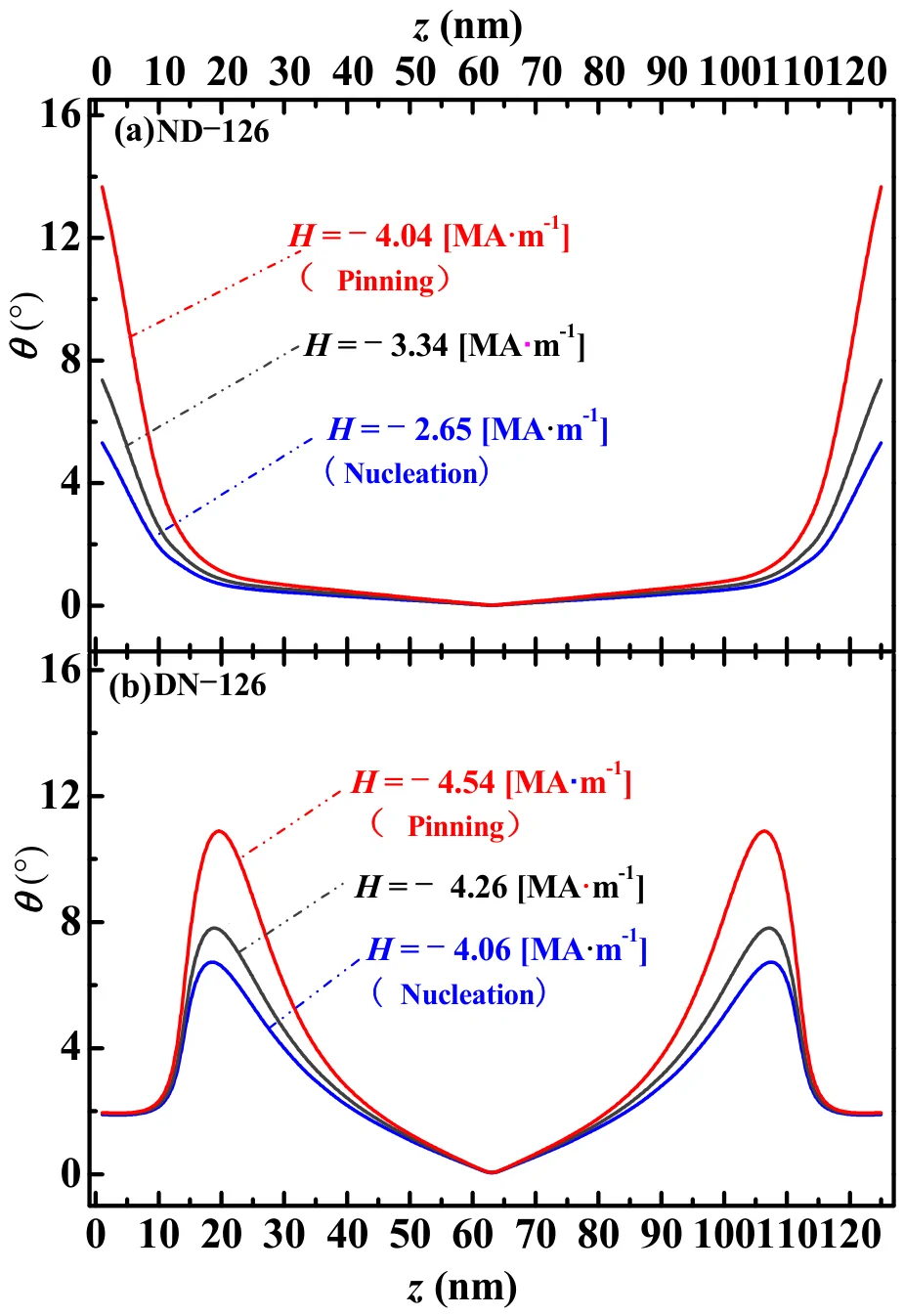
(color online): Angular distributions of Nd_2_Fe_14_B/Dy_2_Fe_14_B nested cubic magnet structures with their easy axes aligned along the *z*-axis: (**a**) ND-126 and (**b**) DN-126.

Figure 8 presents the angular distributions of Nd_2_Fe_14_B/Dy_2_Fe_14_B exchange-coupled magnets throughout the nucleation-to-pinning process, where the outer and inner side lengths are 126 nm and 100 nm, respectively. According to Figure 8a,b, the complete ND-126 and DN-126 magnets maintain two and six magnetic domains, respectively. Owing to the structural symmetry of the magnets, only half of the cubic region along the z-direction (*z* = 0–63 nm) is analyzed below. This region corresponds to one and three magnetic domains in the ND-126 and DN-126 magnets, respectively. Figure 8a shows the angular distribution of the DN-126 magnet, where the Nd_2_Fe_14_B phase is located in the outer region. At the nucleation point *H* = −*H_N_* = −2.65 MA·m^−1^, the angular difference Δ*θ* is 5.31°. With decreasing applied field, Δ*θ* increases to 7.35° at *H* = −3.34 MA·m^−1^ and further to 13.66° at the pinning point *H* = −*H_P_* = −4.04 MA·m^−1^, while spatial domain size Δ*z* remains constant at 62 nm. Figure 8b shows the angular distribution of the DN-126 magnet, where the Dy_2_Fe_14_B phase is located in the outer region. For the ND-126 magnet, the angular difference Δ*θ* evolves from the nucleation to the pinning. At the nucleation point *H* = −*H_N_* = −4.06 MA·m^−1^, the Δ*θ* values at different regions are 0.01°, 4.91°, and 6.79°, respectively. As the applied field decreases to *H* = −4.26 MA·m^−1^, these values evolve to 0.01°, 5.99°, and 7.90°, respectively. Finally, at the pinning point *H* = −*H_P_* = −4.54 MA·m^−1^, Δ*θ* further increases to 0.02°, 9.07°, and 11.01°, respectively. Meanwhile, the corresponding spatial domain sizes Δ*z* remain unchanged at 2, 16, and 44 nm, respectively, and the spatial positions of these domains remain unchanged throughout the magnetization reversal process.

### 3.3. Demagnetization Processes of Nd_2_Fe_14_B/Dy_2_Fe_14_B Magnets with Different Geometries

**Figure 9:**
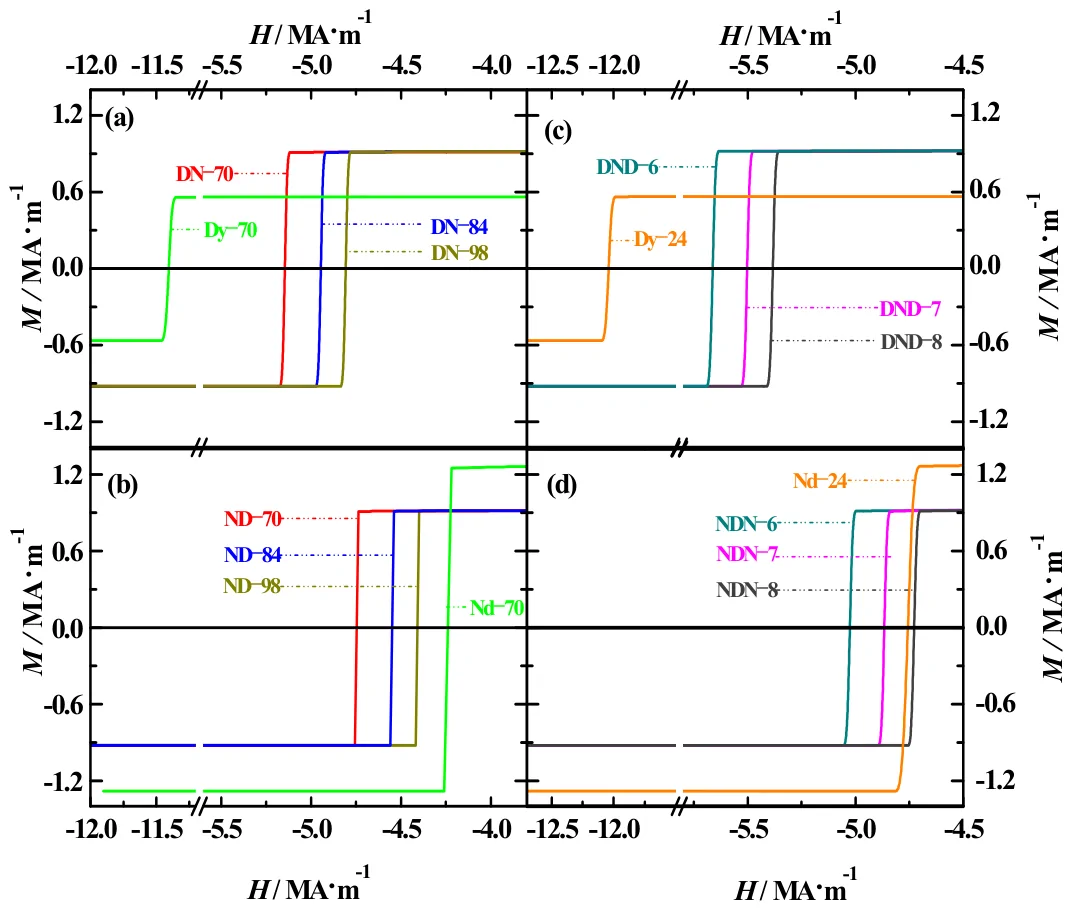
(color online): Demagnetization curves of Nd_2_Fe_14_B/Dy_2_Fe_14_B exchange-coupled nested cylindrical and sandwich magnet structures with their easy axes aligned along the *z*-axis. Nested cylindrical structures: (**a**) Dy_2_Fe_14_B surrounding Nd_2_Fe_14_B (DN); (**b**) Nd_2_Fe_14_B surrounding Dy_2_Fe_14_B (ND). Sandwich structures: (**c**) Dy_2_Fe_14_B enclosing Nd_2_Fe_14_B (DND); (**d**) Nd_2_Fe_14_B enclosing Dy_2_Fe_14_B (NDN).

To examine whether the phase-distribution-dependent magnetic behavior is applicable to different geometrical configurations with the same size fraction of the two main phases, nested cylindrical Nd_2_Fe_14_B/Dy_2_Fe_14_B exchange-coupled magnets are constructed using MuMax3. Meanwhile, cubic sandwich-structured models are further established using OOMMF to verify the consistency and universality of the results obtained from different micromagnetic simulation platforms. Figure 9 presents the demagnetization curves of Nd_2_Fe_14_B/Dy_2_Fe_14_B magnets with their easy axes aligned along the *z*-axis. For the nested cylindrical magnets, the outer diameter (axial length) and inner diameter (axial length) are set to 70 nm (30 nm)/60 nm (20 nm), 84 nm (42 nm)/72 nm (28 nm), and 98 nm (54 nm)/84 nm (36 nm), respectively (Figure 9a,b). For the sandwich-structured magnets, the thicknesses of the three layers from bottom to top are set to 6 nm/12 nm/6 nm, 7 nm/14 nm/7 nm, and 8 nm/16 nm/8 nm, respectively (Figure 9c,d). To elucidate the effect of replacing Nd with Dy in Nd_2_Fe_14_B on the magnetic properties, the dual-main-phase structures are compared with single-phase Nd_2_Fe_14_B and Dy_2_Fe_14_B reference magnets having identical geometrical dimensions. The diameter (axial length) of the single-phase cylindrical magnets is set to 70 nm (30 nm), while the side length of the single-phase cubic sandwich-structured magnets is set to 24 nm. The geometric parameters and sample IDs of the investigated structures are provided in Table 2. The nucleation field *H_N_*, pinning field *H_P_*, and coercivity *H_C_* extracted from the demagnetization curves are summarized in Table 5. For all investigated structures, *H_C_* is equal to *H_P_*, indicating that the coercivity mechanism is governed by domain-wall pinning.

As shown in Table 5, regardless of the geometrical configuration and the micromagnetic simulation software employed, the Nd_2_Fe_14_B/Dy_2_Fe_14_B exchange-coupled magnets exhibit the same trend. With identical volume fractions of the two main phases and the same magnet size, the configuration with Nd_2_Fe_14_B located inside the Dy_2_Fe_14_B phase exhibits a higher critical field than the inversely arranged configuration. Moreover, the critical field decreases with increasing magnet size, which is consistent with the behavior observed for the inner–outer cubic structures discussed in Section 3.2.1. The qualitatively consistent trends identified in these two sections are also observed in this section through the comparison of the critical fields of single-phase and dual-main-phase magnets with different structural configurations, as well as those of the inner–outer cubic structures discussed in Section 3.2.1. In this section, only the nested cylindrical magnets are taken as an example to discuss the effects of phase distribution and magnet size on the magnetic properties of Nd_2_Fe_14_B/Dy_2_Fe_14_B magnets based on specific numerical data. When the outer diameter increases from 70 nm to 98 nm, *H_N_* and *H_C_* of the DN configuration decrease from 4.74 MA·m^−1^ and 5.14 MA·m^−1^ to 4.08 MA·m^−1^ and 4.80 MA·m^−1^, respectively. The ND configuration exhibits the same trend, with *H_N_* and *H_C_* decreasing from 3.44 MA·m^−1^ and 4.74 MA·m^−1^ to 2.55 MA·m^−1^ and 4.40 MA·m^−1^, respectively. Under the same size, the DN configuration exhibits higher critical fields than the ND configuration.

### 3.4. Discussion

To further examine whether the effects of phase spatial distribution and magnet size on the critical fields of dual-main-phase exchange-coupled magnets composed of two single-phase magnets with different magnetocrystalline anisotropy fields *H_A_* follow the same qualitative trends as those observed in Nd_2_Fe_14_B/Dy_2_Fe_14_B exchange-coupled magnets. Two additional dual-main-phase systems, Nd_2_Fe_14_B/La_2_Fe_14_B and Nd_2_Fe_14_B/SmCo, are investigated using nested cubic structures using MuMax3. To isolate the effect of main-phase composition on the magnetic properties of dual-main-phase magnets, the same nested cubic geometry as that used for the Nd_2_Fe_14_B/Dy_2_Fe_14_B magnets (Figure 1b) is adopted for both systems. The sample IDs are summarized in Table 2. The mesh size and the selection criteria for the other simulation parameters are kept identical to those used for the Nd_2_Fe_14_B/Dy_2_Fe_14_B nested cubic magnets [39]. The calculation results indicate that all investigated magnets exhibit a pinning-controlled coercivity mechanism. For the Nd_2_Fe_14_B/La_2_Fe_14_B exchange-coupled magnets, the *H_N_* of NL-70, NL-126, and NL-180 are 1.23, 1.00, and 0.72 MA·m^−1^, respectively, while their *H_C_* are 1.63, 1.33, and 1.19 MA·m^−1^, respectively. In contrast, the *H_N_* values of the LN-70, LN-126, and LN-180 exchange-coupled magnets are −1.17, −1.41, and −1.53 MA·m^−1^, respectively, with corresponding *H_C_* values of 0.94, 0.78, and 0.72 MA·m^−1^, respectively. For the nested cubic Nd_2_Fe_14_B/SmCo exchange-coupled magnets, the *H_N_* values of SN-70, SN-126, and SN-180 are 4.28, 4.08, and 3.40 MA·m^−1^, respectively, and the corresponding *H_C_* values are 5.00, 4.58, and 4.40 MA·m^−1^, respectively. For the corresponding NS-70, NS-126, and NS-180 configurations, the *H_N_* values are 3.09, 2.65, and 2.63 MA·m^−1^, respectively, while the *H_C_* values are 4.38, 4.04, and 3.90 MA·m^−1^, respectively. The calculated results for the Nd_2_Fe_14_B/La_2_Fe_14_B and Nd_2_Fe_14_B/SmCo exchange-coupled magnets show that both *H_N_* and *H_C_* decrease with increasing magnet size. More importantly, under the same geometrical dimensions, both single-phase and dual-main-phase magnets with the magnetic phase possessing a higher *H_A_* exhibit higher *H_N_* and *H_C_*. The above results indicate that, for dual-main-phase magnets with identical phase fractions and geometries, placing the phase with the higher *H_A_* in the outer region is sufficient to achieve higher critical fields, consistent with the results obtained for the Nd_2_Fe_14_B/Dy_2_Fe_14_B system.

## 4. Conclusions

In this work, micromagnetic simulations based on MuMax3 and OOMMF are employed to systematically investigate the magnetization reversal behavior and coercivity mechanisms of single-phase Nd_2_Fe_14_B, Dy_2_Fe_14_B, and Nd_2_Fe_14_B/Dy_2_Fe_14_B exchange-coupled magnets with different main-phase distributions. The results demonstrate that, under identical geometrical conditions, Dy_2_Fe_14_B magnets exhibit higher nucleation fields *H_N_* and coercivities *H_C_*, which are closely related to the larger magnetocrystalline anisotropy field *H_A_* of Dy_2_Fe_14_B. For the 80 nm cubic magnets, the *H_N_* and *H_C_* values of Dy_2_Fe_14_B are 10.71 and 11.11 MA·m^−1^, respectively, compared with 2.17 and 4.02 MA·m^−1^ for Nd_2_Fe_14_B. The larger *H_A_* corresponds to a greater resistance to magnetic-moment deviation from the easy-axis direction, thereby enhancing the resistance to magnetization reversal. With increasing magnet size, both the *H_N_* and *H_C_* of single-phase and dual-main-phase magnets decrease. For the nested cubic Nd_2_Fe_14_B/Dy_2_Fe_14_B exchange-coupled magnets with Dy_2_Fe_14_B as the outer phase, *H_N_* decreases from 4.16 to 3.36 MA·m^−1^, while *H_C_* decreases from 4.96 to 4.38 MA·m^−1^ as the outer side length increases from 70 to 180 nm. For exchange-coupled Nd_2_Fe_14_B/Dy_2_Fe_14_B magnets with identical phase fractions, placing the Dy_2_Fe_14_B phase with a higher *H_A_* in the outer region leads to enhanced *H_N_* and *H_C_*. For nested cubic Nd_2_Fe_14_B/Dy_2_Fe_14_B exchange-coupled magnets with an outer side length of 70 nm, placing Dy_2_Fe_14_B in the outer region and Nd_2_Fe_14_B in the inner region results in a *H_N_* of 4.16 MA·m^−1^ and a *H_C_* of 4.96 MA·m^−1^, whereas the reverse phase arrangement, with Nd_2_Fe_14_B in the outer region and Dy_2_Fe_14_B in the inner region, results in *H_N_* of 3.07 MA·m^−1^ and *H_C_* of 4.38 MA·m^−1^. This enhancement originates from the fact that the outer Dy_2_Fe_14_B phase provides a more stable outer region with higher reversal resistance, thereby delaying the propagation of reversed domains and increasing the critical field for irreversible reversal. The evolution of in-plane magnetic moments and angular distributions further reveals that the initial deviation of magnetic moments preferentially occurs in the Nd_2_Fe_14_B region, which possesses a lower magnetocrystalline anisotropy field than Dy_2_Fe_14_B. Furthermore, the dependence of *H_N_* and *H_C_* on phase distribution is also obtained in Nd_2_Fe_14_B/La_2_Fe_14_B and Nd_2_Fe_14_B/SmCo exchange-coupled magnets. In both systems, placing the phase with the higher *H_A_* in the outer region resulted in higher *H_N_* and *H_C_* than the reverse phase arrangement. These results indicate that the effect of placing the high-*H_A_* phase in the outer region is not limited to the Nd_2_Fe_14_B/Dy_2_Fe_14_B exchange-coupled system and may provide a broadly applicable design consideration for regulating coercivity in dual-main-phase permanent magnets.

## Figures and Tables

**Table 1:** Intrinsic magnetic properties of the four magnetic materials. Here, *A*, *K*, *M_S_*, *H_A_*, *δ*, and *l_ex_* represent the exchange constant, magnetocrystalline anisotropy constant, saturation magnetization, magnetocrystalline anisotropy field, Bloch wall width, and magnetostatic exchange length, respectively.

Materials	*A*/J·m^−1^	*K*/J·m^−3^	*M_S_*/A·m^−1^	*H_A_*/MA·m^−1^	*δ*/nm	*l_ex_*
Nd_2_Fe_14_B [26,27,28,29]	7.70 × 10^−12^	4.30 × 10^6^	1.28 × 10^6^	5.35	4.20	2.74
Dy_2_Fe_14_B [26]	7.90 × 10^−12^	4.24 × 10^6^	5.65 × 10^5^	11.95	4.29	6.28
La_2_Fe_14_B [26]	7.00 × 10^−12^	1.10 × 10^6^	1.10 × 10^6^	1.59	7.92	3.03
SmCo [27,28,29]	1.20 × 10^−11^	5.00 × 10^6^	5.50 × 10^5^	14.48	4.86	7.95

**Table 2:** Model types, materials, shapes, parameters, sizes and sample IDs of the simulated single-phase and dual-main-phase magnets.

Model Types	Materials	Shapes	Parameters	Sizes/nm	Sample IDs
single-phase	Nd_2_Fe_14_B or Dy_2_Fe_14_B	cubic	*t* in Figure 1a	70	Nd-70
					Dy-70
				80	Nd-80
					Dy-80
				90	Nd-90
					Dy-90
		cylindrical	*t*_1_ (*l*_1_) in Figure 1c	70 (30)	Nd-70
					Dy-70
		sandwich	*t*_1_/*t*_2_/*t*_1_ in Figure 1d	6/12/6	Nd-24
					Dy-24
dual-main-phase	Nd_2_Fe_14_B/Dy_2_Fe_14_B	nested cubic	*t*_1_ (*t*_2_) in Figure 1b	70 (55.5)	ND-70
					DN-70
				126 (100)	ND-126
					DN-126
				180 (142)	ND-180
					DN-180
		nested cylindrical	*t*_1_ (*l*_1_)/*t*_2_ (*l*_2_) in Figure 1c	70 (30)/60 (20)	ND-70
					DN-70
				84 (42)/72 (28)	ND-84
					DN-84
				98 (54)/84 (36)	ND-98
					DN-98
		sandwich	*t*_1_/*t*_2_/*t*_1_ in Figure 1d	6/12/6	NDN-6
					DND-6
				7/14/7	NDN-7
					DND-7
				8/16/8	NDN-8
					DND-8
	Nd_2_Fe_14_B/La_2_Fe_14_B	nested cubic	*t*_1_ (*t*_2_) in Figure 1b	70 (55.5)	NL-70
					LN-70
				126 (100)	NL-126
					LN-126
				180 (142)	NL-180
					LN-180
	Nd_2_Fe_14_B/SmCo			70 (55.5)	NS-70
					SN-70
				126 (100)	NS-126
					SN-126
				180 (142)	NS-180
					SN-180

**Table 3:** Nucleation field *H_N_*, coercivity *H_C_*, pinning field *H_P_* of single-phase Nd_2_Fe_14_B and Dy_2_Fe_14_B cubic magnets with the easy axes aligned along the *z*-axis.

Materials	*H_N_*/MA·m^−1^	*H_C_* (*H_P_*)/MA·m^−1^
Nd-70	2.21	4.08
Dy-70	10.79	11.15
Nd-80	2.17	4.02
Dy-80	10.71	11.11
Nd-90	1.61	3.96
Dy-90	10.59	11.07

**Table 4:** Nucleation field *H_N_*, coercivity *H_C_*, and pinning field *H_P_* of Nd_2_Fe_14_B/Dy_2_Fe_14_B nested cubic exchange-coupled magnets with their easy axes aligned along the *z*-axis.

Materials	ND-70	DN-70	ND-126	DN-126	ND-180	DN-180
*H_N_*/MA·m^−1^	3.07	4.16	2.65	4.06	2.61	3.36
*H_C_* (=*H_P_*)/MA·m^−1^	4.38	4.96	4.04	4.54	3.88	4.38

**Table 5:** Nucleation field *H_N_*, coercivity *H_C_* and pinning field *H_P_* of two-phase Nd_2_Fe_14_B/Dy_2_Fe_14_B nested cylindrical and sandwich magnet structures with their easy axes aligned along the *z*-axis.

Structure	Materials	*H_N_*/MA·m^−1^	*H_C_* (*H_P_*)/MA·m^−1^
cylindrical	Nd-70	2.07	4.22
	Dy-70	11.07	11.39
	ND-70	3.44	4.74
	DN-70	4.74	5.14
	ND-84	2.85	4.54
	DN-84	4.30	4.94
	ND-98	2.55	4.4
	DN-98	4.08	4.80
Sandwich	Nd-24	3.14	4.74
	Dy-24	11.74	12.02
	NDN-6	4.00	5.02
	DND-6	5.63	5.65
	NDN-7	3.70	4.86
	DND-7	5.27	5.49
	NDN-8	3.44	4.72
	DND-8	5.06	5.37

## Data Availability

The data presented in this study are available on request from the corresponding author.
